# Lack of K13 mutations in *Plasmodium falciparum* persisting after artemisinin combination therapy treatment of Kenyan children

**DOI:** 10.1186/s12936-016-1095-y

**Published:** 2016-01-22

**Authors:** Julian Muwanguzi, Gisela Henriques, Patrick Sawa, Teun Bousema, Colin J. Sutherland, Khalid B. Beshir

**Affiliations:** Department of Immunology and Infection, London School of Hygiene and Tropical Medicine, London, UK; Human Health Division, International Centre of Insect Physiology and Ecology, Mbita Point, Western Kenya Kenya; Department of Medical Microbiology, Radboud University Nijmegen Medical Centre, Nijmegen, The Netherlands

**Keywords:** *k13*-*propeller*, Artemisinin resistance, Western Kenya, Africa, Slow clearance, Sub-microscopic, qPCR

## Abstract

**Background:**

Studies in Southeast Asia reported a strong relationship between polymorphisms at the propeller domain of the Kelch 13 (K13) protein encoded by the *Plasmodiumfalciparumk13(pfk13)* gene and delayed parasite clearance after artemisinin treatment. In Africa, *P. falciparum* remains susceptible and combination therapy regimens which include an artemisinin component display good efficacy. Using quantitative real-time PCR (qPCR), sub-microscopic persistence of *P. falciparum* has previously been reported in one-third of children treated with artemisinin combination therapy (ACT) in western Kenya. In this study, further investigation was made to evaluate whether these sub-microscopic residual parasites also harbour mutations at the propeller region of *pfk13* and whether the mutations, if any, affect treatment outcome.

**Methods:**

The *pfk13* propeller domain was genotyped in DNA samples obtained in 2009 from Kenyan children treated with artemether–lumefantrine (AL) and dihydroartemisinin–piperaquine (DP). Paired samples at pre-treatment (day 0) and day of treatment failure (day 28 or 42) for 32 patients with documented recurrent parasitaemia were available for genotyping. Additional day 3 DNA samples were available for 10 patients.

**Results:**

No mutation associated with artemisinin resistance in Southeast Asia was observed. Only one DP-treated patient harboured a non-synonymous mutation at codon 578 (A578S) of *pfk13*-*propeller* gene in the day 0 sample, but this allele was replaced by the wild-type (A578) form on day 3 and on the day of recurrent parasitaemia. The mutation at amino acid codon 578 showed no association with any phenotype. Polymorphisms in *pfk13* were not responsible for parasite persistence and gametocyte carriage in the children treated with ACT.

**Conclusion:**

This study contributes to the ongoing surveillance of suspected artemisinin resistance parasites in Africa by providing baseline prevalence of *k13*-propeller mutations in western Kenya with samples collected from a longitudinal study.

*Clinical Trials Registration* NCT00868465.

## Background

Artemisinin combination therapy (ACT) is widely used to treat uncomplicated *Plasmodium falciparum* malaria in endemic areas. Significantly reduced susceptibility to artemisinins, characterized by delayed parasite clearance in vivo and enhanced survival of early stage parasites in vitro, has been reported in Southeast Asia [1, 2]. Most recently, these parasite phenotypes have been associated with mutations in the *pfk13* gene of *P. falciparum,* encoding the kelch-domain protein K13 [3]. Further studies reported direct evidence of resistance to artemisinin using genetic modification of different *pfk13* loci [4]. This work has led to an operational definition of partial artemisinin resistance: ≥5 % of patients carrying K13 resistance-associated mutations, all of whom have been found to have either persistent parasitaemia by microscopy on day 3, or a parasite clearance half-life of ≥5 h after treatment with ACT or artesunate monotherapy [5].

In Africa, ACT is the first-line anti-malarial treatment and remains efficacious and safe [3, 6]. However, resistance to previous generations of anti-malarial drugs such as chloroquine, sulfadoxine-pyrimethamine (SP) emerged in the 1970s in Southeast Asia and eventually spread to the Indian sub-continent and then to Africa [7]. It is, therefore, critical that continuous monitoring of the therapeutic response of ACT is carried out in endemic areas in order to detect early warning signs and effectively track and contain the development and spread of artemisinin resistance. Currently, delayed clearance of microscopically detectable parasites has not been observed in Africa. In addition, there are limited data about the in vitro susceptibility of ring stage of parasite development against artemisinins as well as the presence and prevalence of *pfk13* mutations [8]. Recently, a cross-sectional survey was carried out to determine the prevalence of *pfk13* mutations in 14 sub-Saharan African countries. The study reported the absence of mutations that were associated with artemisinin resistance in Southeast Asia, with the exception of codon 543 [9]. In previous study, the presence of residual sub-microscopic *P. falciparum* parasites in western Kenyan children on day 3 after ACT treatment was reported and this was associated with subsequent recrudescence and transmission [10]. In addition, the authors have shown in the same Kenyan patient that *P. falciparum* parasites carrying certain genotypes at *pfmdr1, pfcrt, pfubp1*, and *pfap2mu* genes were found to survive more often after ACT treatment at sub-microscopic level [11]. The aim of this study was to investigate whether these sub-microscopic residual parasites also harboured mutations at the propeller region of *pfk13* and whether the mutations, if any, affect treatment outcome.

## Methods

DNA samples were previously obtained from filter-paper blood spots from children (age 8 months–10 years) treated with either artemether–lumefantrine (AL) or dihydroartemisinin–piperaquine (DP) in a clinical trial carried out in 2009 in Mbita, western Kenya and fully described elsewhere [6, 10]. The study was carried out 5 years after Kenya had officially changed the first line of treatment from SP to AL [12]. The protocol was approved by the Kenya Medical Research Institute Ethical Review Committee and the London School of Hygiene and Tropical Medicine Ethics Committee (reference 5455). Written informed consent was obtained from a parent or guardian of each participating child. This is an ancillary study of *pfk13* propeller domain polymorphism in DNA samples available from the 2009 study at the following time points: for 32 patients with documented recurrent parasitaemia, paired samples at day 0 (pretreatment) and day of treatment failure (28 or 42, PCR-uncorrected); for 10 patients, additional day 3 DNA samples (Table 1).

**Table 1:** Sample availability for *k13* genotyping

Sample ID	D0	D1	D2	D3	D28	D42
K0025	X					X
K0344	X					X
K0385	X					X
K0544	X					X
K0598	X				X	
K0701	X			X		X
K0719	X			X		X
K0774	X					X
K0804	X					X
K0840	X				X	
K0875	X					X
K0881	X					X
K1010	X					X
K1149	X			X		X
K1152	X				X	
K1307	X				X	
K1348	X			X		X
K1368	X	X	X	X		X
K1438	X			X		X
K1478	X				X	
K1521	X					X
K1861	X			X		X
K1917	X				X	
K1943	X					X
K2023	X					X
K2024	X					X
K3077	X					X
K3212	X			X	X	
K3215	X			X		X
K3404	X			X	X	
K3762	X				X	
K3801	X				X	

Table shows sample which were available for *k13* genotyping on different time points. Days 0 and day of fail (days 28 or 42) samples were available for *k13* genotyping for 32 patients. Of the 32, ten patients had additional samples on day 3 while for patient K3168, additional samples on days 1 and 2 were included retrospectively for further *k13* genotype analysis

The K13-propeller gene fragment (coordinates 1726169–1726997 on chromosome 13 of 3D7 isolate (PF3D7_1343700) was amplified by nested PCR using previously published primers [13]. For the first round of PCR, 250 nM primer (Eurofins, Germany), 1X hot fire pol^®^ blend master mix (Solis Biodyne, Estonia) and 5 μl of DNA in a total volume of 20 µl was run under the following cycling conditions: 95 °C for 15 min then 30 cycles at 95 °C for 30 s, 58 °C for 2 min and extension at 72 °C for 2 min and final extension at 72 °C for 10 min. For the second round of PCR, a total volume of 25 μl made of 18.75 μl nuclease free water, 250 nM primer, 1X hot fire pol^®^ blend master mix (Solis Biodyne, Estonia) final concentration and 5 μl of DNA was run under the following cycling conditions: 95 °C for 15 min then 40 cycles at 95 °C for 30 s, 60 °C for 1 min and extension at 72 °C for 1 min and final extension at 72 °C for 10 min. A positive control (K13_5) with one of the mutations identified in Cambodia (provided by D Ménard) was included in every experiment.

Polymorphisms at *pfk13* were determined by direct sequencing of amplicons using ABI BigDye Terminator v3.1 cycling sequencing kit and analysis on an ABI 3730 sequencer (Applied Biosystems, USA) as described previously [11] and chromatogram sequences were analysed using Geneious v6.1.5 (Biomatters, USA) in comparison to *pfk13*-*propeller* sequence region of 3D7 isolate (PF3D7_134700).

## Results

**Fig. 1:**
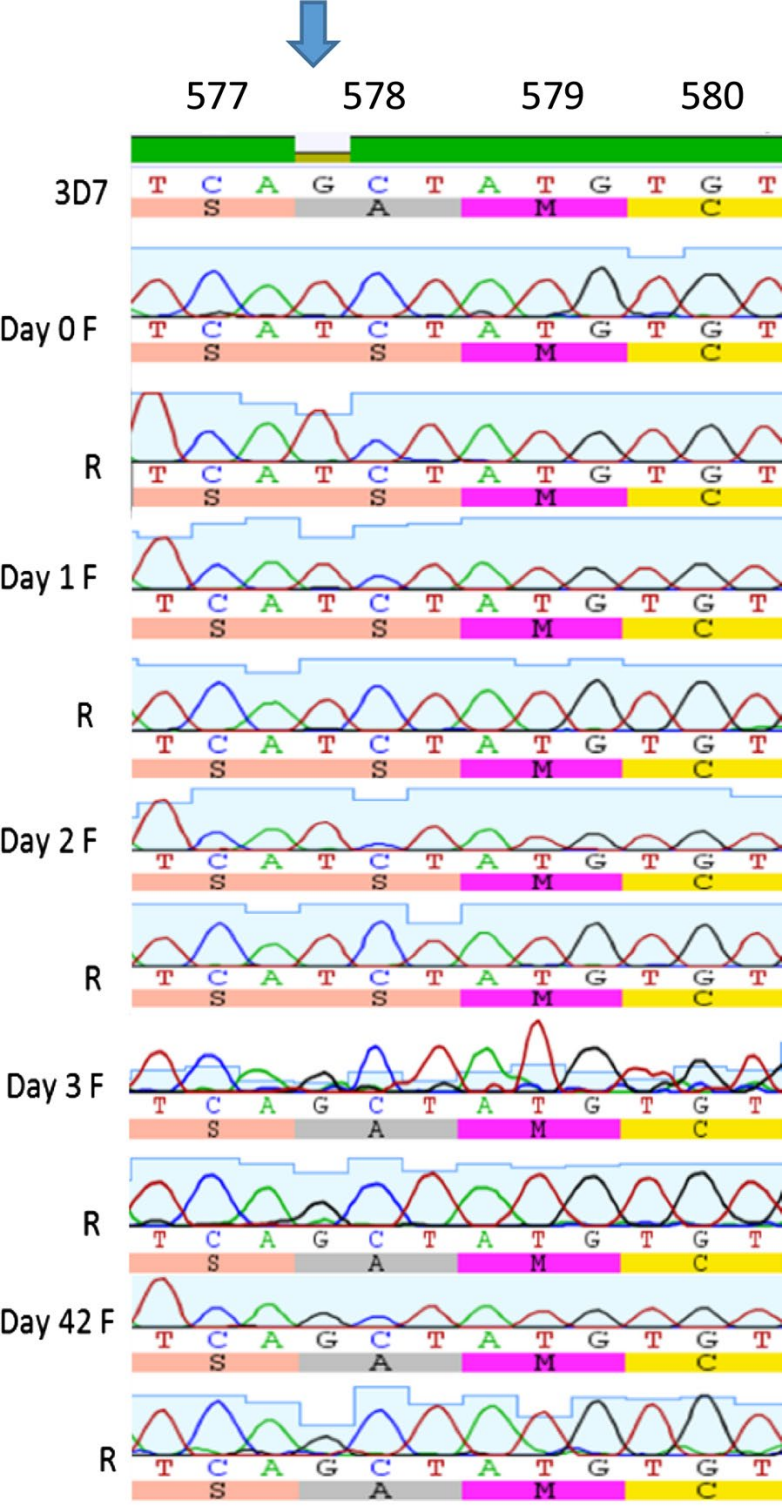
DNA and amino acid sequence of a propeller region of *PfK13* showing mutation at codon 578. Sequence of samples from patient K1368 at day 0, 1 and 2 show mutation at amino acid position 578 while the samples on day 3 and day 42 have no mutations. DNA amplification of sample on day 3 using *pfK13* [13] and *pgmet* primers [18] repeatedly failed. DNA and amino acid sequence of 3D7 isolate was used as a reference. Each sample has a sequence using forward (F) and reverse (R) primers. *Arrow* indicates mutation and *top numbers* indicate amino acid position

*Pfk13* sequences for 32 samples on day 0, seven samples on day 3 and 30 samples on day of failure were successfully obtained and analysed for sequence variation. Of these 69 sequenced isolates, 68 harboured *pfk13* loci encoding propeller domains identical to the reference. A single DP-treated patient (K1368) harboured a non-synonymous mutation at codon 578 of *pfk13*-*propeller* gene in the day 0 sample, but this allele was replaced by the wild-type form on day 3 and on the day of recurrent parasitaemia (day of failure) (Table 2). The K13-propeller sequence was deposited in Gene Bank (accession number KT261646). To understand the clearance dynamics of the mutant parasites in the patient, *pfk13* was retrospectively sequenced in day 1 and day 2 parasites from this individual. Sequence analysis of day 0, day 1 and day 2 samples revealed the presence of mutation at codon A578S, suggesting that the mutant parasite was present 2 days after DP treatment (Figure 1). The treatment outcome and other phenotype and genotype data for the patient are found in Table 2. The patient cleared parasites as measured by microscopy on day 1 after DP treatment and no gametocytes were detected on day 7. Recurrent parasitaemia was observed on day 42 but was classified as new infection after PCR-correction. One additional patient carried a synonymous mutation at *pfk13* codon P553P on day of failure but that was not observed on day 0 or day 3.

**Table 2:** Phenotype and genotype outcomes of patient K1368

Patient ID	Phenotype				
	D3 Parasite positive	Gametocyte D7 positivity	Mosquito infection	Recurrent parasitaemia	PCR correction
K1368	No	No	No	Day 42	New infection

Patient ID	Genotype				
	D0 *Pfk13* at 578	D3 *Pfk13* at 578	D0 *Pfmdr1* at 86/184/1246	Day 0 Pfcrt at 72–76	D0 *PfAP2mu* S160 N
K1368	S	A	NFD	CVMNK	S

The patient phenotypic data show typical normal clearing parasites and all samples before treatment show the wild type alleles with the exception of *pfk13,* where the mutant is present at codon 578. However, 3 days after treatment only the wild type of codon 578 was observed. When the pfk13 genotyping on day 3 was repeated no amplification was observed

## Discussion

No evidence was found that artemisinin resistance-associated mutations in the *pfk13*-encoded propeller domain, including the major resistance-associated mutation C580Y, contribute to ACT treatment failure in Mbita, western Kenya. Only one of the 32 patients from this longitudinal study had mutation in the propeller region of *pfk13* gene, despite evidence of parasite persistence in over 30 % of children [10]. One patient transiently carried parasites harbouring mutation at codon 578 (A578S) on enrolment, and on days 1 and 2 after DP treatment. However, these parasites were apparently cleared by DP in this individual, and persistent parasites detected at day 3 and day 42 harboured the wild type A578 allele only. However, it is not clear whether the wild type was already present on days 0, 1 and 2 but below the detection limit of the assay. The importance of multiplicity of infection was discussed in detail in previous work and the findings broadly agree with Farnert et al., who also showed that there were genetic changes in samples collected in the same patient as short as 6 h apart [10, 14]. In fact, the *msp1* and *msp2* genotyping of the day 0 sample showed that the patient was infected with at least three different parasite clones. Blood from this patient was presented to *Anopheles gambiae* mosquitoes at day 7 by membrane feeding, as reported in previous study, but no infected mosquitoes were generated [6].

The A578S polymorphism has previously been reported in a cross-sectional study in western Kenya, Kisumu, and in four other African countries [9], but the current study is the first study to investigate the role of this variant in gametocyte carriage and infectiousness to mosquito. These findings are consistent with recent published multisite studies in Southeast Asia and Africa that showed mutation at codon 578 in *pfk13* and its lack of association with parasite clearance half-life in patients treated with ACT [3]. In Bangladesh, the mutation at codon 578 has also been observed in clinical isolates and using computational modelling, the authors suggested that it has an effect on tertiary structure of the protein [15]. Although the parasite harbouring the mutant A578S allele was successfully cleared by DP, the contribution of piperaquine in clearing these parasites is not clear. This polymorphism lies adjacent to C580Y, and further in vivo and in vitro studies to clarify the significance of the A578S mutation are warranted, particularly in different genetic backgrounds. Mutations in *pfcrt, pfmdr1, pfap2mu*, and *pfubp1* also contribute to treatment outcomes after either artemisinin monotherapy or ACT treatment [11, 16]. Studies introducing transgenic variants of *pfk13* and other candidate loci into parasite isolates with an African genetic background could clarify whether *pfk13* mutations or polymorphisms in other genes are most relevant in determining artemisinin sensitivity in clinical isolates in Africa [4, 17].

The rarity of *pfk13* variants in the current small study precludes from making meaningful assessment of the role of A578S and other propeller domain mutations in treatment outcomes in Africa. However, it can be confirmed that the sub-microscopic clearance phenotype observed in western Kenya is not directly related to the parasite clearance half-life observed in Southeast Asia. The sub-microscopic residual parasites have been shown previously to be important phenotypes as children harbouring those parasites were significantly more likely to be infectious to mosquitoes and were more likely to have recurrent asexual parasitaemia on day 28 or 42 [10]. Factors associated with the host, drug and parasite should be investigated to determine the exact cause of the sub-microscopic residual parasites.

This study contributes to the ongoing surveillance of suspected artemisinin resistance parasites in Africa by providing baseline prevalence of *k13*-propeller mutations in western Kenya with samples collected from a longitudinal study, and thus one of the first to relate genotype and phenotype. This information from samples collected in 2009 does not reflect the current status of ACT, nor the prevalence of k13-propeller mutations now. Therefore, continuous monitoring of ACT in western Kenya and the collation of phenotypic and genotypic data to track the emergence and spread of parasites with reduced susceptibility to artemisinins is recommend.

## Abbreviations

ACT: artemisinin combination therapy
AL: artemether–lumefantrine
DP: dihydroartemisinin–piperaquine
EDCTP: European and developing countries clinical trials partnership
*pfk13*: *Plasmodium falciparum kelch 13* gene
qPCR: quantitative (real-time) PCR
